# Minimal clinically important difference for the Mandarin version of the Tinnitus Questionnaire determined via anchor-based and distribution-based methods

**DOI:** 10.1186/s12955-022-02072-z

**Published:** 2022-11-30

**Authors:** Hailing Gu, Cong Diao, Jiatong Tang, Bo Yang, Jinfeng Ji, Mengyun Zhou, Zhaoli Meng

**Affiliations:** 1 Department of Otolaryngology-Head and Neck Surgery, Hearing Center/Hearing and Speech Science Laboratory, West China Hospital of Sichuan University, 37 Guo Xue Lane, Chengdu, 610041 Sichuan People’s Republic of China; 2 West China Hospital/West China Medical School, Sichuan University, Chengdu, Sichuan People’s Republic of China

**Keywords:** Mandarin Tinnitus Questionnaire (MTQ), Minimal clinically important difference (MCID), Tinnitus

## Abstract

**Background:**

The previous study showed that the Mandarin Tinnitus Questionnaire (MTQ) has satisfactory reliability and validity. We have also completed the classification of the severity of tinnitus based on MTQ scores. In clinical studies, efficacy is often judged by whether results are statistically significant; however, statistical significance does not necessarily equate to clinical significance, whereas the minimum clinically important difference (MCID) of the scale does. In the following project, we will explore the MCID of the MTQ.

**Methods:**

We recruited participants aged 18 years and above who sought treatment for primary or secondary tinnitus at the Otorhinolaryngology Department of the Hearing Center of West China Hospital, Sichuan University from September 2020 to September 2021. The participants had to undergo the following four assessments of tinnitus severity: doctor evaluation, self-report, the MTQ, and the visual analog scale (VAS), all at baseline and at the follow-up. The MCIDs of the MTQ were established via anchor-based and distribution-based methods. The anchor method used the VAS and self-reported clinical impression as anchors and defined the treatment effectiveness by mean/median and receiver operating characteristic (ROC) curve, while methods of effect size (ES), standard error of measurement (SEM), and reliability change index (RCI) were used in distribution-based methods.

**Results:**

A total of 115 patients were investigated in this study, 57.4% of whom were women. The average age was 43.2 ± 13.20 years. The average MTQ and VAS scores at baseline were 31.3 ± 14.90 and 5.03 ± 2.24, respectively, while the average MTQ and VAS scores at follow-up were 15.9 ± 11.70 and 3.58 ± 2.48, respectively. Moreover, in terms of self-reported clinical impressions, 19 patients indicated that they were cured (16.5%), 24 that it was much better (20.9%), 63 that there was no change (54.8%), and 9 that it was much worse (7.8%). The MCIDs for the change in total MTQ ranged from 6.29 to 19.00, those for improvement from 1.09 to 22.75, and those for deterioration from 3.50 to 7.64.

**Conclusion:**

We selected an absolute value of 7.5 as the MCID for the MTQ score. An increase in MTQ score more than 7.5 was considered aggravation of tinnitus, and a decrease in MTQ score more than 7.5 was considered a reduction in tinnitus.

## Background

The global prevalence of tinnitus is approximately 14%, and more than 2% of people suffer from severe tinnitus [1], with 7.1% actively seeking medical attention and 2.5% requiring clinical intervention [2]. Tinnitus can cause a range of issues, including increased stress, anxiety, depression, sleep disorders, difficulty concentrating, and hearing impairment [3]. Approximately 26% of patients with tinnitus are affected by anxiety [4], 48% to 60% by sadness [5], and 76% by insomnia [6].

Subjective tinnitus can only be quantified indirectly since subjective tinnitus-induced suffering cannot be satisfactorily represented by psychoacoustic parameters (e.g., tinnitus loudness) [7]. Pinto et al. [8] reviewed 16 papers on the most prevalent psychiatric diagnostic criteria and measures of tinnitus annoyance and concluded that psychological diseases, tinnitus severity, and tinnitus distress in patients are all significantly associated. Tinnitus severity and tinnitus distress are strongly related to mental disease. As a result, a variety of tinnitus self-report questionnaires are available to assess the intensity of tinnitus by questioning patients about psychological disorders such as depression, anxiety, and stress to help explain the distress produced by tinnitus [9, 10].

The tinnitus questionnaire (TQ) was one of the first to be developed and the most commonly used [11]. It was developed by Hallam in 1987 and contains 52 items in five categories, namely emotional and cognitive stress, intrusiveness, auditory perceptual difficulties (APDs), somatic complaints, and sleep disturbance [12]. The original English version of the TQ has high internal consistency and reliability [12]. The TQ has been translated into German, Spanish, French, Dutch, Cantonese, and Mandarin [13]. The various translated versions have been widely used in the clinical setting, and their internal consistency and retest reliability have been demonstrated [13–16].

The Mandarin Tinnitus Questionnaire (MTQ) is a Mandarin version of the TQ derived via exploratory factor analysis [13]. It includes 37 questions each in five dimensions: cognitive distress, emotional distress, APDs, intrusiveness, and sleep disturbance. The MTQ and the English, German, and Cantonese TQs have consistent reliability and validity [13]. Logistic regression analysis resulted in the following classification of the severity of tinnitus based on MTQ scores: no tinnitus (a score of < 21), mild tinnitus (21–36), moderate tinnitus (37–47), and severe tinnitus (> 47) [17].

In clinical studies, efficacy is often judged by whether results are statistically significant; however, statistical significance does not necessarily equate to clinical significance, whereas the minimum clinically important difference (MCID) of the scale does [18, 19]. Thus, the MCID of the MTQ should be analyzed, and such studies are rare. Adamchic et al. [20] showed a 5-point decrease in the MCID for improvement and a 1-point increase in the MCID for worsening of the TQ score. Hall et al. [21] recommended using at least the median MCID of 12 (as determined in their study) to indicate a clinically meaningful change in the German TQ score. As different language versions of the TQ contain different numbers of items, the MCID should be determined for each.

In summary, there are few studies regarding efficacy assessment and determination of the MCID of different translated versions of the TQ, including the MTQ. We aimed to fill this clinical gap by analyzing the MCID of the MTQ. Our results may guide the scientific design of clinical treatment plans for patients with tinnitus.

## Methods

### Participants

In this study, we recruited participants aged 18 years and above who sought treatment for primary or secondary tinnitus at the Otorhinolaryngology Department of the Hearing Center of West China Hospital, Sichuan University from September 2020 to September 2021. Patients who were unable to complete the relevant assessment owing to cognitive impairment or difficulty in understanding, psychiatric disorders, or auditory hallucinations were excluded. This research was approved by the Biomedical Research Ethics Committee of West China Hospital (No. 2020 [311]). Participants were enrolled after they provided written informed consent.

### Study design

We collected the demographic information (name, sex, age, telephone number), medical history (side of tinnitus), and tinnitus assessment data (assessment date and four assessment outcomes) of each participant at baseline. After 6 months, we followed them up telephonically. The different types of treatment interventions that participants underwent in different study centers were collected, and their self-reported clinical impression after treatment was recorded, as follows: cure/much better/no change/much worse. In addition, the participants had to undergo the following four assessments of tinnitus severity: doctor evaluation, self-report, the MTQ, and the visual analog scale (VAS), all at baseline and at the follow-up. The doctor's evaluation and the self-report were carried out independently.

#### Doctor evaluation and self-report

First, the doctors asked all patients the following five questions: (1) Do you feel anxious or nervous because of tinnitus? (2) Do you have difficulty in listening to others because of tinnitus? (3) Do you feel that you can never get away from tinnitus? (4) Is sleeping a problem because of tinnitus? (5) Does tinnitus result in headache, ear pain, or tension in the muscles of the head? Based on the answers to these five questions, the patients' tinnitus severity was classified as none, slight, mild, or severe. These five questions were designed to be analogous to emotional distress, auditory perceptual difficulties, cognitive distress, sleep disturbance, and intrusiveness [2]. Second, patients were asked to self-report on their current tinnitus severity according to the same four levels: none, slight, mild, and severe. The doctor’s evaluation and self-report were performed on the same day.

#### MTQ

The MTQ is a self-administered scale that consists of 37 questions, and the total score ranges from 0 (no distress) to 74 (very severe distress) [17]. This questionnaire indicates the degree of tinnitus-related psychopathological symptoms. According to the total MTQ score, patients are divided into four distress levels: none (0–20), mild (21–36), moderate (37–46), and severe (47–74). A higher score indicates a higher degree of tinnitus-induced distress.

#### VAS

The VAS is a very simple, subjective, psychometric response scale. The participants answered it last so that the results of the MTQ would not be influenced by any tiredness that the participants felt. In this study, patients conveyed their tinnitus-induced distress by indicating a position along a line marked 0 to 10. Zero indicated that the patient was not distressed, while 10 indicated that the patient was very severely distressed.

### Statistical analysis

All continuous variables are presented as means (standard deviations [SDs]), and categorical data are presented as counts (percentages). Student’s t test or one-way analysis of variance was used for the comparison of continuous variables among groups, and the chi-squared test or Fisher’s exact test was used for the comparison of categorical variables between groups, as appropriate. Two regression models were used for flexibility in examining associations between the exposure and each outcome. The change in MTQ score from baseline was defined as ΔMTQ. An improvement in the MTQ score indicated a reduction in tinnitus severity (including much better, ΔVAS ≥ 1), while deterioration indicated exacerbation of tinnitus (including much worse, ΔVAS ≤ − 1).

There is no standardized method to determine the ideal MCID. However, methodologists generally recommend triangulating the results of multiple methods [21]. In this study, the MCIDs of the MTQ were established via anchor-based and distribution-based methods.

#### MCIDs determined via an anchor-based method

MCID calculations should be based on patient-reported outcomes, e.g., the VAS score and self-reported clinical impression, which are correlated at r ≥ 0.30–0.35 and consist of appropriate patient-based and clinical anchors [22]. Thus, we calculated the Spearman rank correlation of self-reported clinical impression with the ΔMTQ scores and the Pearson correlation of the VAS score with the MTQ scores at baseline and at 6 months. These two methods were used as anchors to define the treatment effectiveness. Patients who differed by at least one point on the VAS at baseline and at 6 months were censored. The ΔMTQ score was calculated, and the mean (for normally distributed variables) or median (for non-normally distributed variables) absolute ΔMTQ score was recorded as the MCID. The VAS and self-reported clinical impression were used as the main anchors, and a receiver operating characteristic (ROC) curve was used to determine the optimal MCID cut-off point for the MTQ score.

#### MCIDs determined via a distribution-based method

A distribution-based approach was used to calculate the magnitude of small, intermediate, and large domain score differences [23]. On the basis of benchmark effect sizes (ESs) determined in a previous study (0.2, small; 0.5, intermediate; and 0.8 or greater, large) [24], small, intermediate, and large domain score differences in MTQ scores were calculated via the following equation:

$${\text{ES}} = ({\text{MTQ}}_{{{\text{baseline}}}} { } - {\text{MTQ}}_{{\text{follow - up}}} )/{\text{SD}}_{{{\text{baseline}}}} .$$

In this equation, SD is the standard deviation of the baseline MTQ score. Domain score differences calculated with the above equation were compared with the observed differences in mean MTQ scores between the different clinical anchor states [25].

The standard error of measurement for the MTQ score was also computed, as follows:

$${\text{SEM}} = {\text{SD}}_{baseline} \sqrt {1 - \alpha }$$

where $\alpha$ is the test–retest reliability of the MTQ, i.e., 0.93 [13]. We calculated 1.96 SEMs as an estimate of the MCID to reduce the probability of false positive results [26].

$${\text{RCI}} = \sqrt {2SEM^{2} }$$

where the reliable change index (RCI) is the change in MTQ score divided by the square root of the SEM [27]. The RCI is an expression of the change in score in SD units, much like a z score. Therefore, we set this equal to 1.96 (the value on a standard normal curve associated with a 95% confidence interval) according to Beaton et al. [28], and solved for the change score in the numerator, to give the minimum change in MTQ score considered significantly different to no change at all (at *p* < 0.05).

#### Statistical software

All statistical tests were two-sided, and *p* < 0.05 was regarded as significant. All statistical analyses were performed by using open-source statistical analysis software (R version 4.0.5; The R Foundation for Statistical Computing, Vienna, Austria), IBM SPSS Statistics for Windows version 25 (IBM Corp., Armonk, New York, USA), and GraphPad Prism 8.3.0 software (GraphPad Software, San Diego, California, USA).

## Results

### Sociodemographic and clinical characteristics of patients with tinnitus

**Fig. 1:**
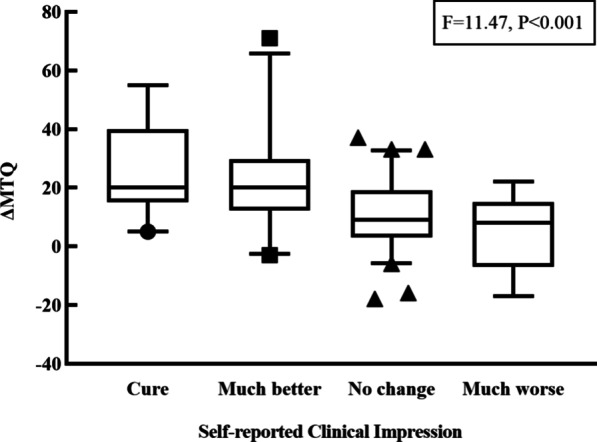
Boxplots of ΔMTQ score in four groups categorized according to the self-reported clinical impression. ΔMTQ from baseline were determined by subtracting the value at follow-up from the baseline value. MTQ, Mandarin Tinnitus Questionnaire

A total of 115 patients were investigated in this study, 57.4% of whom were women (Table 1). The average age was 43.2 ± 13.20 years. Among the patients, 40.0% complained of bilateral tinnitus, 33.0% of left-sided tinnitus, and 27.0% of right-sided tinnitus. The doctor-evaluated tinnitus severity was mostly slight, accounting for 69.6% at baseline and 60.9% at the follow-up. Nineteen patients self-reported that their tinnitus disappeared by the time of the follow-up. The average MTQ and VAS scores at baseline were 31.3 ± 14.90 and 5.03 ± 2.24, respectively, while the average MTQ and VAS scores at follow-up were 15.9 ± 11.70 and 3.58 ± 2.48, respectively. Moreover, in terms of self-reported clinical impressions, 19 patients indicated that they were cured (16.5%), 24 that it was much better (20.9%), 63 that there was no change (54.8%), and 9 that it was much worse (7.8%). The distributions of ΔMTQ scores in the four self-reported clinical impression groups (F = 11.47, *p* < 0.001) are illustrated in Fig. 1, including the median value and 5th–95th percentile.

**Table 1:** Sociodemographic and clinical characteristics of the patients. (N = 115)

Characteristics	N (%)	Characteristics	N (%)
Sex		Laterality	
Female	66 (57.4%)	Bilateral	46 (40.0%)
Male	49 (42.6%)	Left	38 (33.0%)
Age (mean ± SD)	43.2 ± 13.20	Right	31 (27.0%)
Doctor-evaluation tinnitus severity at baseline		Doctor-evaluation tinnitus severity at the follow-up	
Severe	1 (0.8%)	Severe	1 (0.9%)
Mild	34 (29.6%)	Mild	25 (21.7%)
Slight	80 (69.6%)	Slight	70 (60.9%)
None	0 (0.0%)	None	19 (16.5%)
Self-report tinnitus severity at baseline		Self-report tinnitus severity at the follow-up	
Severe	17 (14.8%)	Severe	7 (6.1%)
Mild	47 (40.9%)	Mild	41 (35.7%)
Slight	51 (44.3%)	Slight	48 (41.7%)
None	0 (0.0%)	None	19 (16.5%)
		Self-reported clinical impression	
MTQ at baseline (mean ± SD)	31.3 ± 14.90	Cure	19 (16.5%)
VAS at baseline (mean ± SD)	5.03 ± 2.24	Much better	24 (20.9%)
MTQ at the follow-up (mean ± SD)	15.9 ± 11.70	No change	63 (54.8%)
VAS at the follow-up (mean ± SD)	3.58 ± 2.48	Much worse	9 (7.8%)

MTQ, Mandarin version of the Tinnitus Questionnaire; VAS, visual analogue scale

### MCIDs determined via the anchor-based method

The correlation coefficients between the self-reported clinical impression and ΔMTQ (r_1_), and VAS score and MTQ score at baseline (r_2_) were calculated using the self-reported clinical impression and VAS score as the main anchors. The correlation coefficients were as follows: r_1_ = 0.453 (*p* < 0.001) and r_2_ = 0.619 (*p* < 0.001).

According to the change in self-reported clinical impression, 24 patients reported improvement and 9 reported deterioration; for these changes, the MCID values were 22.75 and 3.63, respectively. Twenty-eight patients changed one point on the VAS, and 65 patients changed at least one point on the VAS; their MCID values were 9.39 and 25.50, respectively. The mean and SD of the difference between the scores of MTQ under the two anchors were calculated, and the mean of the difference was recorded as the MCID (Table 2).

**Table 2:** The MCID of MTQ for tinnitus patients determined by the subjective criteria in anchor methods

Anchor Methods	Subjective Criteria	N	MCID (Mean/Median)
*Improvement*			
Self-report change impression	Much better	24	22.75
ΔVAS	= 1	20	7.50
	> 1	51	32.25
*Deterioration*			
Self-report change impression	Much worse	9	3.63
ΔVAS	= − 1	8	3.50
	< − 1	14	4.50
*Change*			
Self-report change impression	Much better/much worse	33	19.00
ΔVAS	= ± 1	28	9.39
	> 1 or < − 1	65	25.50

MCID, minimal clinically important difference; ΔVAS, VAS change from baseline was determined by subtracting the value at visit from baseline value

The results of the MCID for the MTQ score according to the ROC analyses are summarized in Table 3. The cut-off point for deterioration based on self-reported impression of change was 13.5, which corresponded to an area under the ROC curve of 0.775.

**Table 3:** MCID for MTQ according to the ROC analyses

Subjective criteria	N	MCID	AUC (95% CI)	Sensitivity	Specificity	+ LR	− LR
*Improvement*							
Self-report change impression	24	14.5	0.704 (0.577, 0.708)	0.708	0.365	1.940	0.459
ΔVAS	71	− 3.5	0.605 (0.407, 0.803)	0.905	0.667	1.357	0.286
*Deterioration*							
Self-report change impression	9	13.5	0.775 (0.675, 0.876)	0.718	0.273	2.634	0.387
ΔVAS	22	6.5	0.602 (0.430, 0.775)	0.636	0.364	1.750	0.571

MCID, minimal clinically important difference; CI, confidence interval; + LR, positive likelihood ratio; − -LR, negative likelihood ratio; ΔVAS, VAS change from baseline was determined by subtracting the value at visit from baseline value

### MCIDs determined via distribution-based methods

Distribution-based methods are used to estimate the MCID based on the observed distribution of score changes [29]. The results of the MCID for the MTQ score are presented in Tables 4 and 5, which were calculated by three variation indexes on two subjective criteria: the ES, SEM, and RCI. When ES = 0.5, the MCID values for improvement, deterioration, and total change according to self-reported clinical impression were 8.21, 7.64, and 8.07, respectively, and those for the VAS score were 1.09, 7.64, and 6.29, respectively (Table 4). Similarly, the MCIDs calculated for the MTQ score when 1.96SEM was used as the intermediary index according to self-reported clinical impression were 8.52, 7.93, and 8.37, respectively, and those calculated according to the VAS score were 1.13, 7.93, and 6.53, respectively (Table 5). The MCIDs calculated for the total when 1.96RCI was used as the intermediary index according to self-reported clinical impression were 12.04, 11,21, and 11.83, respectively, and those calculated according to the VAS score were 1.60, 11.21, and 9.23, respectively (Table 5).

**Table 4:** The MCID value of MTQ determined by ES

Subjective criteria		n	SD_baseline_	ES = 0.2	ES = 0.5	ES = 0.8
*Improvement*						
Self-report change impression	Much better	24	16.42	3.28	8.21	13.14
ΔVAS	≥ 1	71	2.18	3.06	1.09	12.23
*Deterioration*						
Self-report change impression	Much worse	9	15.29	0.44	7.64	1.74
ΔVAS	≤ − 1	22	15.29	3.06	7.64	12.23
*Change*						
Self-report change impression		33	16.13	3.23	8.07	12.91
ΔVAS		93	12.59	2.52	6.29	10.07

MCID, minimal clinically important difference; ES, effect size

**Table 5:** The MCID value of MTQ was determined by SEM and RCI

Subjective criteria		SEM	1.96SEM	RCI	1.96*RCI
*Improvement*					
Self-report change impression	Much better	4.34	8.52	6.14	12.04
ΔVAS	≥ 1	0.58	1.13	0.81	1.60
*Deterioration*					
Self-report change impression	Much worse	4.05	7.93	5.72	11.21
ΔVAS	≤ − 1	4.04	7.93	5.72	11.21
*Change*					
Self-report change impression		4.27	8.37	6.04	11.83
ΔVAS		3.33	6.53	4.71	9.23

MCID, minimal clinically important difference; SEM, standard error of measurement; RCI, reliability change index

### Results of various approaches combined

**Fig. 2:**
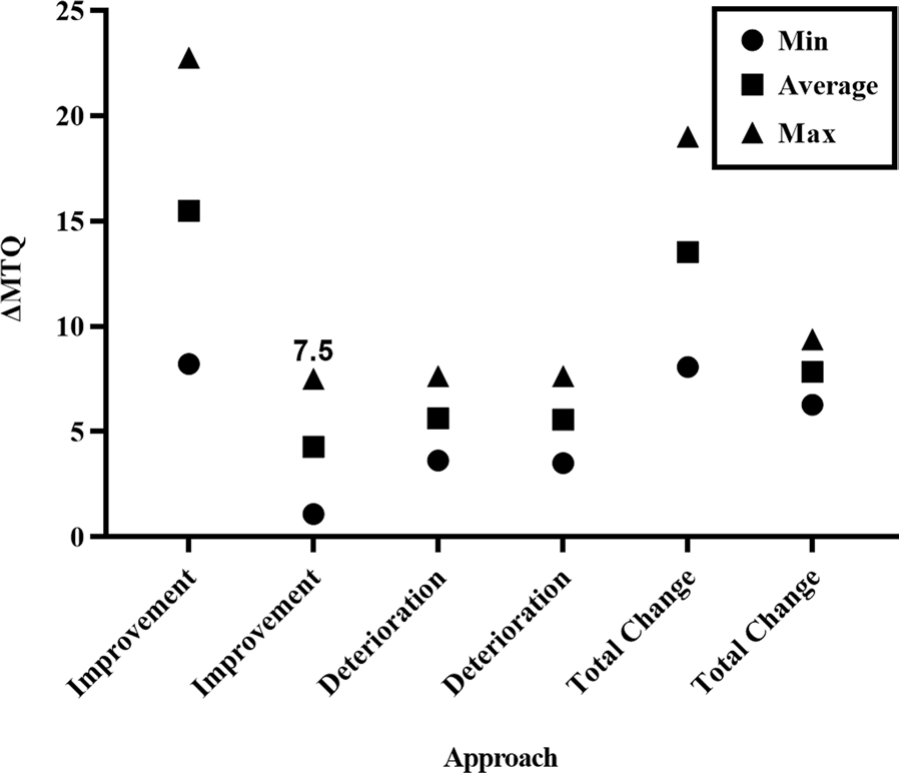
Summary of distribution and anchor based estimates of the MCID. MTQ, Mandarin Tinnitus Questionnaire; MCID, minimal clinically important difference

The MCIDs for the change in total MTQ ranged from 6.29 to 19.00, those for improvement from 1.09 to 22.75, and those for deterioration from 3.50 to 7.64 (Table 6 and Fig. 2).

**Table 6:** MCID for the MTQ, derived by various approaches

Subjective criteria	Anchor-based		Distribution-based			MCID		
	Mean/Median	ROC	0.5ES	1.96SEM	1.96RCI	Min	Average	Max
*Improvement*								
Self-report change impression	22.75	14.5	8.21	8.52	12.04	8.21	15.48	22.75
ΔVAS	7.50	− 3.5	1.09	1.13	1.60	1.09	4.29	7.50
*Deterioration*								
Self-report change impression	3.63	13.5	7.64	7.93	11.21	3.63	5.63	7.64
ΔVAS	3.50	6.5	7.64	7.93	11.21	3.50	5.57	7.64
*Total change*								
Self-report change impression	19.00		8.07	8.37	11.83	8.07	13.53	19.00
ΔVAS	9.39		6.29	6.53	9.23	6.29	7.84	9.39

MCID, minimal clinically important difference; ROC, receiver operating characteristic; ES, effect size; SEM, standard error of measurement; RCI, reliability change index

## Discussion

The MCID reflects the change in a score sufficient to indicate an impact of clinical treatment on a patient, and its primary function is to help clinical and research staff determine whether statistically significant score changes on a scale are clinically meaningful. In this study, we used both anchor-based and distribution-based methods to analyze the MCID of the MTQ to determine the smallest score change that was both statistically and clinically significant.

### Selection of anchors in the anchoring method

Through correlation analysis, we discovered that self-reported clinical impression and VAS scores correlated best with changes in MTQ scores (r > 0.6), while physician evaluations correlated poorly with changes in MTQ scores (r < 0.6). This may be because the MTQ is a patient self-assessment scale, similar to the VAS and different to physicians' evaluations. This result emphasizes that the MTQ score accurately reflects patients' tinnitus.

### Determination of the MCID value

The VAS and self-reported clinical impression were selected as anchors for analysis, and the mean/median and ROC curve were used for analysis and calculation of the MCID, respectively. The distribution-based method is used to estimate the MCID based on the observed distribution of score changes. Different MCID values were calculated (Table 6). The next objective was to determine the clinically most appropriate MCID values from the many calculated MCID values. We did this by comparing the results obtained via several analysis methods. We tried to minimize the placebo effect (which has a prevalence of up to 40% in tinnitus treatment [30]) by selecting the maximum MCID value as cut-off.

Considering that self-reported clinical impression is a qualitative variable and the VAS score is a quantitative variable, the VAS score more closely correlates with MTQ score changes. The MCID value for MTQ score improvement (7.50) calculated with the VAS score as anchor was similar to the MCID value for deterioration (7.64). Therefore, we decided to use the MCID value calculated with the VAS score as anchor. After treatment, the MTQ score of patients with tinnitus decreased by 7.5 or more than that before treatment. This result suggests that the treatment is effective in resolving or ameliorating tinnitus. Similarly, when the MTQ score increased by 7.5 or more compared with pretreatment, the patient’s tinnitus was considered aggravated.

In their 2012 MCID study on the TQ, Adamchic et al. [20] determined an MCID of − 5 and + 1 for the TQ, by using the Clinical Global Impression score as the ROC method in the anchoring method for calculation, taking response bias into account. In their 2018 study of the MCID for the German version of the TQ, Hall et al. [21] suggested a median of 12 as the MCID value, considering measurement bias and error. Therefore, we selected two subjective indicators with good correlations as anchors for the analysis and used the VAS score calculated based on the results as anchor.

A major limitation of this study was the following. For patients with tinnitus whose initial MTQ score was less than 7.5, we could not judge whether the treatment they received was effective in terms of the MCID. However, according to our previous study, a score of less than 21 on the MTQ is considered to indicate no problem with tinnitus [17]. When the initial score of the patient is less than 7.5, we believe that tinnitus has little impact on their quality of life. The next step is to expand the sample size in future studies to verify the adaptability of the MTQ in terms of treatment-related changes.

## Conclusion

The change in MTQ score can be used as a clinical index to quantify the efficacy of tinnitus treatment and can be put into use in domestic studies related to tinnitus interventions, but physicians need to use it in conjunction with the severity of tinnitus experienced by the patient. The selected MCID for the MTQ score was an absolute value of 7.5. An increase in MTQ score more than 7.5 was considered aggravation of tinnitus, and a decrease in MTQ score more than 7.5 was considered a reduction in tinnitus.

The MCID of the MTQ score can guide the design of personalized clinical treatment plans for patients with tinnitus. The MTQ has been tested for reliability and validity and used for classification of tinnitus severity. Our results may assist in the development of relevant tinnitus questionnaires and guide their clinical use in China.

## Data Availability

The datasets used and/or analysed during the current study are available from the corresponding author on reasonable request.

## Abbreviations

ES: Effect size
MCID: Minimal clinically important difference
MTQ: Mandarin Tinnitus Questionnaire
RCI: Reliability change index
ROC: Receiver operating characteristic
SEM: Standard error of measurement
TEQ: Tinnitus evaluation questionnaire
THI: Tinnitus handicap inventory
THI-CM: Mandarin Chinese tinnitus handicap inventory
THQ: Tinnitus handicap questionnaire
TQ: Tinnitus questionnaire
TRQ: Tinnitus reaction questionnaire
VAS: Visual analogue scale
